# The cooperative coaching client: Clients’ responding activities to wh-questions for solution generation in coaching

**DOI:** 10.1177/14614456261418103

**Published:** 2026-03-21

**Authors:** Melanie Fleischhacker, Eva-Maria Graf, Sanna Vehviläinen

**Affiliations:** 1 University of Klagenfurt, Austria; 2 University of Eastern Finland, Finland

**Keywords:** questions for solution generation, wh-questions, clients’ responding activities, client cooperativeness, conversation analysis, business coaching

## Abstract

While solution generation and question-answer sequences are key to change in coaching, little is known about the role of questions with a solution-oriented agenda. Even less research exists on clients’ (cooperative) responding activities. To breach this gap, we used Conversation Analysis to investigate responses to wh-questions for solution generation with a special focus on the continuum of (dis-)aligning and (dis-)affiliating responses and the degrees of clients’ (un-)cooperativeness. The sequential analysis yielded five response categories: “no(n) answer,” “repair initiation,” “not answering and refocusing,” “answering” and “extended answering.” While the response possibility space includes a range of (un-)cooperative reactions, clients display an overall willingness to answer questions with a solution-generating function: “answering” and “extended answering” make up 68.9% of responses. Additionally, in-depth analyses revealed further cooperative behaviors. Apart from their interactional cooperativeness, this suggests an interaction-type specific readiness of coaching clients to participate in their own change process, that is, an institutional cooperativeness.

## Solution generation via questioning practices in coaching: An introduction

Supporting change is the *raison d’être* of professional helping interactions (Graf and Spranz-Fogasy, 2018; Graf et al., 2019; Scarvaglieri et al., 2022). In its goal-oriented emphasis on clients’ (professional) learning and development, this purpose particularly underlies business coaching (Cox et al., 2023; Schermuly, 2019). In contrast to problem-oriented, often retrospective formats such as psychotherapy, coaching is primarily prospective and solution-focused. It generally assumes clients to be mentally healthy (Graf, 2019: 32) and stable as well as efficient, willing and eager to develop and change (Drath, 2012: 17; Schermuly, 2019: 88). Coaching as “goal-oriented self-reflection” (Greif, 2008) thereby draws on the assumption (originating in solution-focused brief therapy) that problem-talk creates problems and solution-talk creates solutions. As part of the basic activity “Co-Constructing Change” (Graf, 2019), solution generation is thus a central, communicative and interactive goal. Solutions for clients’ concerns are ideally co-constructed by coach and client in a dialogue at eye-level in their process-oriented interaction (Jautz, 2017). Within the overall change project, coaches are assumed to be responsible for the process, clients for introducing the topic(s) and concern(s) (Cox et al., 2023), that is, there is an (idealized) distribution of epistemic and deontic authority. Yet, in steering the process by setting the agenda, promoting self-reflection and finding solutions for clients’ concerns (Jautz et al., 2023), coaches concurrently influence its thematic scope and development.

A case in point here is asking questions. As initiating actions, they set a particular response – also regarding its content – conditionally relevant (Schegloff, 2007) thus allowing coaches to steer the interaction. Considered as the major intervention in coaching according to the vast majority of practice literature (e.g. Schreyögg, 2012; Wehrle, 2024), the omnipresence, omnirelevance and interaction-type specificity of questioning practices were recently confirmed by the linguistic-psychological project “Questioning Sequences in Coaching” (QueSCo) (Graf et al., 2024, 2025b). Given that questions function as building blocks of institutional activities, coaches have a substantial interactional share in generating solutions for and with clients. As Köller (2004) suggests, “questions initiate hypothetical imaginative processes that have an immanent tendency to turn into self-reflection processes” (662). Apart from initiating such processes of (self-)reflection and imagination, which are considered vital for solution generation (Greif, 2008; Moos and Spranz-Fogasy, 2024), questions also foster changes in perspective and generate new insights. They help to (re-)build and (re-)structure information to establish new ways of thinking, experiencing, feeling and acting (Köller, 2004).

While change represents the desired outcome in coaching, little is known how change-inducing practices such as questions (Peräkylä et al., 2008 for psychotherapy) contribute to clients’ learning and development, and how they are responded to and processed on the communicative micro-level. Thus, the “Linguistic Coaching Process Research” (Fleischhacker and Graf, 2024) paradigm set out to investigate coaching as a locally co-constructed interaction and to document and analyze its interventions. The QueSCo project, for instance, identified 12 question types that help fulfill several coaching-specific functions including “solution generation” (Graf et al., 2024). First insights into solution generation indicate that coaches draw on “a wide repertoire of questioning actions to stimulate self-reflection” (Moos and Spranz-Fogasy, 2024: 2) and to support a solution-oriented agenda in general (Fleischhacker et al., 2024; Kabatnik and Graf, 2021). Clients’ reactions, in turn, have so far only been investigated in detail within the context of resisting actions (Dionne et al., 2024) and without particular focus on solution-oriented questioning. Yet, as underlying institutional goals shape question-answer sequences in professional helping contexts (Heritage and Clayman, 2010), the institutional context “coaching” also influences clients’ responding activities here.

To help close this gap, the current paper uses Conversation Analysis (CA) to investigate clients’ immediate responding behavior to a particularly relevant question type in coaching, i.e., questions for solution generation (QfSG). We are particularly interested in the entire “response possibility space” (Stivers, 2022: 22), that is, the different ways in which clients orient to the solution-generating agenda and its constraints and create various levels of (un-)cooperativeness in and through their reactions that may eventually support or impede solution generation. Questions are defined here functionally as predominantly initiating actions that entail a (high degree of) response mobilization and make a reaction conditionally relevant (Schegloff, 2007). While they can take various forms (wh-question, polar interrogative question, alternative question or declarative question (e.g. Sidnell and Stivers, 2013; Spranz-Fogasy, 2020)), only wh-questions are addressed. They are important in helping professions such as coaching (Dionne et al., 2024; Graf et al., 2024) and particularly for solution generation (Fleischhacker et al., 2024). Defined in accordance with Couper-Kuhlen and Selting (2017: 20), “wh-questions are interrogatively marked utterances which make use of ‘question words’ to request specific kinds of information.” A major characteristic is their propitiousness for deeply embedded presuppositions (Clayman and Heritage, 2002: 206). According to MacMartin (2008), this feature makes them a particularly thought-provoking intervention in helping interactions. What is more, as wh-questions are less constraining for clients yet often require multi-unit responses (Thompson et al., 2015), these questions seem fruitful to initiate (self-)reflection and to co-construct learning and development (Köller, 2004: 662), that is, to eventually help generate solutions. For this article, QfSG are defined (drawing on, yet going beyond, the QueSCo project) as initiating actions that implement solution-oriented institutional functions or tasks by inviting clients to envision ideal future scenarios, address their own abilities, strengths and resources, generate concrete solutions and measures or evaluate their current state or change progress (see also 3.2 for more detail).

The article is structured as follows: we first introduce relevant CA concepts (“Researching question-answer sequences and clients’ (un-)cooperativeness (in helping professions)” section) and sum up prior research on solution-oriented questioning in psychotherapy and coaching (“Prior research on (wh-)questions for solution generation in helping professions” section). “Method, data and process” Section details method, data and analytical process. “Findings” section presents the response categories “no(n) answer”, “(other-initiated) repair initiation”, “not answering and refocusing”, “answering” and “extended answering” using illustrative data examples. The final part, “The response possibility space and cooperativeness of coaching clients” section, indicates the response possibility space and addresses the continuum of cooperativeness, relating our findings to underlying institutional expectations and coaching clients’ agency and willingness to change.

## Researching question-answer sequences and clients’ (un-)cooperativeness (in helping professions)

As “powerful tool[s] to control the interaction” (Hayano, 2013: 395) that make an answer conditionally relevant (Schegloff, 2007), questions – and QfSG specifically – set unique constraints on responding activities. QfSG in particular convey optimistic presuppositions, set solution-focused topical/action agendas and preferences thereby implementing a variety of social actions (Clayman and Heritage, 2002; Hayano, 2013; Stivers, 2022). The specific set-up of the question or of a series of questions also carries out or supports a particular course of action, for example, carving out solution strategies (Stivers, 2022).

Wh-questions (also content or open questions; Hayano, 2013; Stivers, 2022) are (most frequently) employed to request pieces of information depending on the question word or phrase used (Couper-Kuhlen and Selting, 2017), for example: *What is the next step towards your goal?* At the same time, their scope (i.e. topical/action agenda) is often relatively broad so that recipients can answer more in their own terms (Hayano, 2013; Stivers, 2022); for instance, in the example, the kinds of steps were not pre-defined. Wh-questions are asked from a low and uninformed epistemic K^-^ position, claiming a lack of knowledge (Heritage, 2012). However, wh-questions contain deeply embedded presuppositions (Clayman and Heritage, 2002) all of which would be confirmed by providing the projected answer. This means that “for question recipients to address a problematic presupposition, they must depart from the action agenda of the question” (Stivers, 2022: 9). MacMartin (2008) thus suggests that wh-questions can be particularly thought-provoking. Wh-questions often make relevant extended, multi-unit responses thereby allowing for ample (self-)reflection. At the same time, refuting or challenging them requires additional interactional work.

While responses are thus always made relevant by (wh-)questions and not providing (an appropriate) answer leads to interactional trouble (Schegloff, 2007), a range of responding possibilities is available to interactants. These possibilities must be viewed in relation to the initiating wh-question’s various constraints, as respondents may resist them in multiple ways thereby claiming epistemic authority, autonomy and control (Hayano, 2013; Stivers, 2022). At the same time, responses should be considered in relation to the full range of more or less appropriate alternative reactions, the “response possibility space” (Stivers, 2022: 3), that speakers may draw on to convey not only information or stances but also manage their relationship. CA commonly describes responding actions via preference organization and type-conformity (Lee, 2013; Schegloff, 2007). In wh-question-answer sequences, a preference for response over no response and non-answer responses (i.e., claiming inability or lack of knowledge or initiating repair) has been documented (Hayano, 2013; Lee, 2013). Additionally, type-conforming responses are preferred over type-nonconforming responses (Hayano, 2013; see also design- and action-based preference) with type-conforming responses providing the answer specified by the question words (Robinson and Bolden, 2010). Yet, wh-questions can be further divided into two types with different interactional consequences or preferences. “Specifying questions” seek a particular kind of information. In this case, phrasal responses do simple answering while (phrase-in-)clausal responses indicate trouble with the question (Fox and Thompson, 2010). “Telling questions,” in turn, seek extended responses such as reports, stories or accounts and thus multi-clausal responses are type-conforming (Thompson et al., 2015).

More recently, CA research on relational aspects in helping interactions has focused on the dimensions of alignment and affiliation to describe adjacency pairs such as (wh-)question-answer sequences (e.g. Muntigl, 2024; Stivers, 2022) and thus responding activities. Due to the strong link between the first and second pair part, such pairs are essential in establishing and managing relations and (un-)cooperativeness (Muntigl, 2024). While alignment refers to formal, structural and actional cooperation allowing for the progressivity of the sequence according to the terms of the question, affiliation is central to relationship-building. Affiliative responses are prosocial supporting the project or agenda underway alongside the other person’s needs. Maximally affiliative responses also cooperate with the preference of the question (Muntigl, 2024; Steensig, 2020; Stivers, 2022). Disaligning and disaffiliative responses, in turn, put stress on the relationship as they do not cooperate with the prior action. Here, the notion of interactional resistance has gained empirical ground, understood as a series of client disaffiliative actions that may lead to alliance ruptures (Muntigl, 2024). While these concepts help to describe respondents’ orientation to wh-questions as initiating action and to what degree they support the ongoing (topical and action) agenda, client cooperation or cooperativeness is as of yet (theoretically and empirically) under-researched and ill-defined in linguistics and CA.

As a first step to gain further insights, in this article, we draw on the concepts of affiliation and alignment in their entire continuum (Stivers, 2022) to investigate and describe coaching clients’ responding activities and their degrees of cooperativeness within these.^1^ Being cooperative here means orienting to the solution-oriented agenda in terms of projected action and content, productively engaging with its optimistic presupposition thereby advancing the goal-oriented interaction and solution-orientation. Best case, this means clients’ responding activities align and affiliate with both the wh-question’s agenda and the underlying solution project. By providing cooperative responses, coaching clients either generate solutions or lay the foundation for further solution-oriented work. However, as with (dis-)aligning and (dis-)affiliative actions, cooperativeness also exists on a continuum, which will also be addressed here.

## Prior research on (wh-)questions for solution generation in helping professions

### Optimistic, solution-oriented and miracle questions in psychotherapy

Drawing on CA, MacMartin (2008) was the first to focus on “optimistic questions” in therapy, which centered on clients’ agency, strengths, abilities and successes and most often took the form of wh-questions. Such questions included, for instance, those that suggest patients to have agency and control, for example: “*How does it feel to sort of see where you have been exerting your influence and having control over him?*” (MacMartin, 2008: 86). Due to their deeply embedded (optimistic) presuppositions (MacMartin, 2008: 80), most questions resulted in interactional trouble. MacMartin discerned two broad categories of disaligning responses: *answer-like responses* in the form of optimistic downgrading (e.g. in response to the question above: “*It feels good, but then I wonder why can’t I apply it to other areas with him*” (p. 86)), refocusing (e.g. from own positive attributes to others) or joking and sarcastic responses (all of which superficially seem to align and affiliate yet are treated as problematic and evasive) and *non-answer responses* (which openly display resistance via an inability or unwillingness to respond, e.g. “*I don’t know*”).

Following McMartin, various studies (Mack et al., 2016; Spranz-Fogasy, 2020; Spranz-Fogasy et al., 2018) focused on the functional question type “solution-oriented question” (SOQ) in psychodiagnostic interactions. Using *linguistische Gesprächsanalyse* (Deppermann, 2008), they were defined as initiating actions that seek (concrete) solutions to problems and concerns and elicit expectations and wishes regarding clients’ personal and/or professional future and the helping interaction itself (Mack et al., 2016). The broad category of SOQs was exemplified with “*How would you like it to be if you could say what you would like to change?*” (Mack et al., 2016: 82) yet also with much more concrete questions such as “*What do you think are ways one can achieve something like that for oneself?*” (Mack et al., 2016: 85). Concurrently, SOQ were found to implement other psychotherapeutic aims such as strengthening clients’ agency, personal responsibility and self-confidence. Most importantly, they allowed therapists to assess clients’ general ability to imagine change or generate solutions (see also Spranz-Fogasy, 2020). Spranz-Fogasy et al. (2018) further analyzed the formal, contextual and sequential characteristics of SOQ. Here, too, wh-questions were the most frequent format. They found SOQ to be prospective, projective and hypothetical and occurring toward the end of sessions or complex thematic activities. Their sequential analysis revealed that SOQ always followed expressions of reduced agency and that clients’ responses were almost always dispreferred and disaligned (see e.g. the response to the first question example “*What should I change about myself? Uhm whether I can ever change the fact that I don’t like being alone, I don’t know*” (Spranz-Fogasy et al., 2018: 126).

In turn, Weatherall and Gibson (2015) used CA to describe the delivery and processing of the miracle question technique in a single solution-based therapy session. This type of question presupposes a problem-free future in which the client has already reached their goal and elicits information on how the client would be able to tell that a miracle had occurred. In a series of question-answer sequences, following the client’s first tentative and hedged response, the therapist increasingly pursues a solution-focused agenda by continually topicalizing and reformulating therapeutically relevant aspects. The technique eventually allows the client “to define a set of behaviors to work toward” (Weatherall and Gibson, 2015: 179).

This prior research illustrates that solution-oriented questions play a role in psychotherapeutic interactions yet seem to pose interactional challenges. Helping professionals need to interactively manage resistance or pursue their solution-oriented agenda across a series of question-answer sequences to receive a satisfactory response. Also, most studies so far have exclusively focused on resistance or interactional trouble rather than client cooperativeness or degrees of alignment/affiliation.

### Questions for solution generation in coaching

Questions in general and QfSG in particular play a central role in “Linguistic Coaching Process Research” (LCPR; Fleischhacker and Graf, 2023, 2024). Two early studies compared certain question types in coaching and psychotherapy. Investigating questions requesting examples, Spranz-Fogasy et al. (2019) found that these questions are rare in coaching because clients frequently volunteer such information. Also, clients display no resistance to illustrating experiences or concerns with meaningful examples. In a second study, Kabatnik and Graf (2021) addressed SOQ, defining them in analogy to Mack et al. (2016) for psychotherapy. Their analysis revealed a higher frequency of SOQ in coaching and, in contrast to psychotherapy, they were found throughout the entire coaching interaction, that is, also at the beginning of processes, sessions and activities. In coaching, SOQ also more concretely and immediately addressed explicit changes. Their central and primary function was (helping) to generate solutions rather than to gain insights into clients’ inner states. However, their centrality alongside the frequency of such questions and the variety of institutional functions they implement called for a differentiation of SOQ^2^ and a (conceptual) departure from psychotherapeutic research.

The interdisciplinary research project “Questioning Sequences in Coaching” (QueSCo) addressed this call for differentiation alongside a more general need to gain systematic insights into authentic questioning practices in coaching (Graf et al., 2023). Using *Linguistische Gesprächsanalyse* and CA, QueSCo developed the first empirical, coaching-specific typologies of questions and questioning sequences and – together with psychology – investigated their occurrences, frequencies and effectiveness. QueSCo evinced a typology of 12 institutional question types attributed to 7 basic functions (Graf et al. 2024, 2025b).

**Figure 1.**
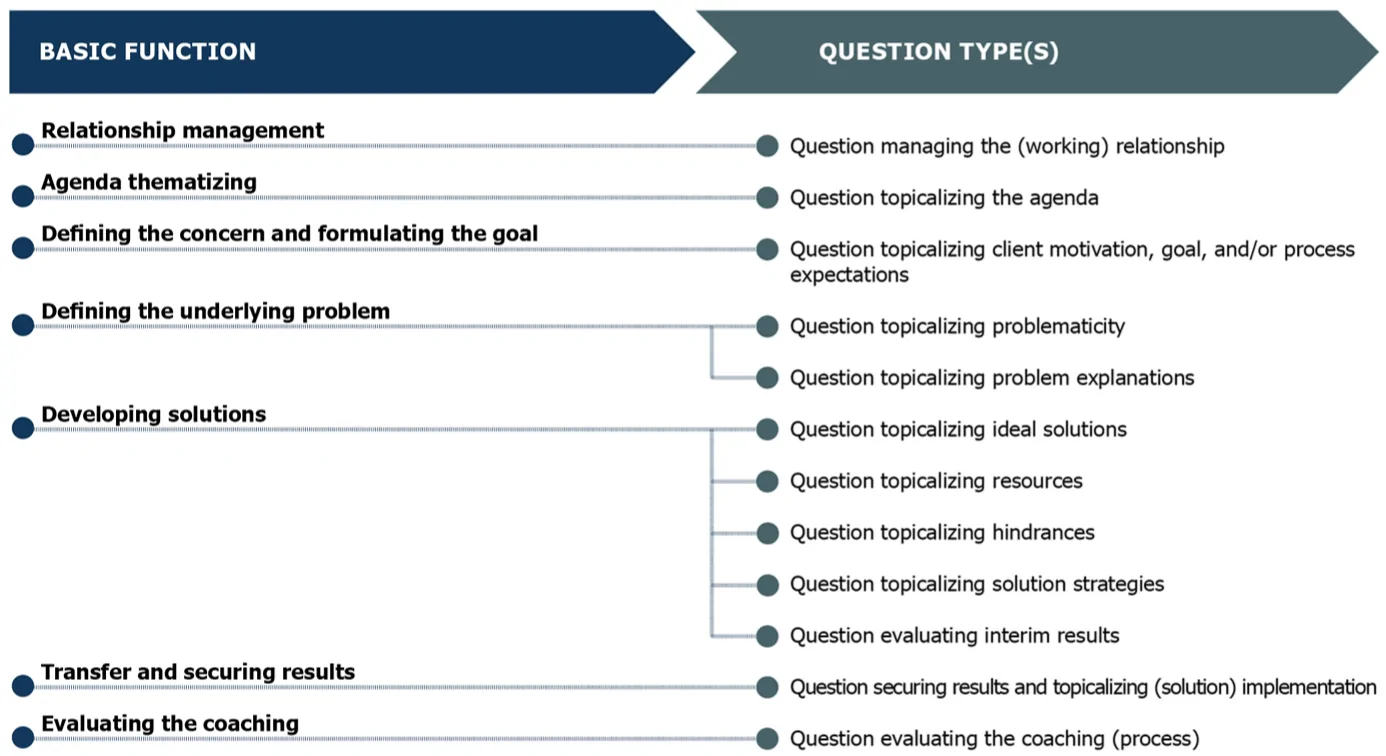
Typology of coaching-specific question types.

As Figure 1 illustrates, five of these question types are dedicated to solution generation, that is, represent QfSG in coaching. More specifically, these question types are initiating actions that aim at carving out ideal solutions, expectations and wishes; addressing and activating resources; dismantling hindrances; detailing concrete steps and measures and establishing a connection between current and envisioned states by evaluating the (change) progress. Table 1 further details the different question types including descriptions and examples based on the QueSCo manual (Graf et al., 2024).

**Table 1.** Questions for solution generation (QfSG) in coaching.

Question type	Description	Example(s)
Questions topicalizing ideal solutions	These questions are future-oriented and often hypothetical. They invite clients to imagine desired changes in their own thinking, feeling or behavior and to articulate their vision of an ideal future, solution or scenario. They help clarify goals, explore possibilities or client aspirations.	(1) *What would be a good result? What would be a desired behavior?* (2) If you imagine in 5 years, we are sitting in the cinema, curtain goes up, and your life comes on. *What do you think we will see there?*
Questions topicalizing resources	These questions focus on identifying and activating clients’ resources – both internal (abilities, strengths) and external (e.g. other people) – that help them solve problems or reach their goal. They make use of existing knowledge, positive experiences or competencies (often by revisiting past events). In some coaching approaches, they explore personality parts as resources. Additionally, they help reveal gaps in resources and encourage clients to consider new perspectives or actions.	(1) *What have you already tried to orient yourself here?* (2) The experiences you’ve had now, despite your doubts in the beginning. *What does that do to you?* *(3) What do you think, who else could help?* (4) *Are those three* (personality parts) *a good team to carry you through this?*
Questions topicalizing hindrances	In contrast to identifying resources, these questions explore obstacles and factors blocking progress in the sense of developing or implementing solutions. These hindrances are “symptoms” or concrete manifestations of problems, problematic behaviors or (external) factors that clients perceive as obstacles and which need to be addressed to generate solutions.	(1) *What prevented that?* (2) *So, there was also a fearful part of you? An anxious part?* (3) And when you say it like that. *How strong is the anxious part still?*
Questions topicalizing solution strategies	Questions on solution strategies help develop, reflect on and practice new or alternative behaviors and ways of thinking or acting. Building on previous insights, these questions guide decisions about next steps and concrete actions. They often address the how, when, where and why.	(1) *What can you do now to use this energy for yourself in such a way that it leads you to your goal?* (2) *What would be your next concrete step?*
Questions evaluating interim results	These questions often close activities and are employed to assess (interim) progress. For this, they may (re-)establish a connection between the solution and the underlying problem or concern, that is, clients’ current/past and envisioned state. The questions help to establish whether the client or the coaching is on track, which changes have occurred and how far goals have been advanced. Additionally, they monitor ongoing progress between sessions, mapping the clients’ current state and development at the beginning of a new session.	(1) *How does this reflection affect you? What does it mean for your decision?* (2) Now, those are the elements that you need. *Do you think that they can be provided in your current job?* (3) *How did you feel after the coaching, after the last session? Is there a different situation than two weeks ago?*

Frequency analyses revealed that QfSG make up 41% of the 3691 questions within the QueSCo corpus of authentic, systemic coaching processes. Another striking result was that clients provided an aligned answer to coaches’ questions in 73% of cases. Wh-questions constituted the most frequent grammatical form (44%; Graf et al., 2025b). Going beyond Kabatnik and Graf’s (2021) findings regarding the occurrence of SOQ across entire coaching sessions/processes, QueSCo calculations determined their distribution along established phases of change (Deplazes et al., 2018; Graf et al., 2020), that is, their placement within the overall structure of coaching (Robinson, 2013: 267). While all types were most frequently used in the phase “Co-constructing Change” (see also the “Basic Activity” in Graf, 2019), they all occurred in “Formulating the Concern,” too. All QfSG were used to different degrees across the various phases.

In their recent article, which further investigated QfSG in coaching, Graf et al. (2025b) also showed how these question types are involved in “supra-sequential” activities (Robinson, 2013: 263) they called “problematizing,” “weighing” and “integrating”. This corroborates yet specifies prior results in the sense that QfSG are used throughout the entire coaching conversation. It highlights the goal- and solution-orientation of the helping format “coaching” in which “solution-orientedness” is expected and continuously oriented to by both coach and client. Finally, Fleischhacker et al.’s (2024) contribution used *Linguistische Gesprächsanalyse* and coding in MAXQDA to further detail the formal, functional, thematic and semantic features of the various question types. They found that QfSG do not only differ in function and thematic focus but also display other diverging characteristics such as tendencies for single or multi-unit questions, hypothetical or factual language, prospective or retrospective orientations and placement in the interaction. Though the study illustrated the various question types in their sequential context, it focused on the questions rather than clients’ responses or whether these cooperate with the solution-oriented agenda of such questions.

Overall, very little research exists on clients’ responding actions in coaching. Winkler (2022) established a coding scheme for semi-responsive answers based on topical (e.g. shifts, expansions) and formal dimensions (e.g. shifts in verb tense). Dionne et al. (2024) investigated instances of resistance following wh-questions. Building on Muntigl (2024), they found that clients in coaching resist by *refusing to answer*, c*omplaining* and *disagreeing with the question’s agenda and presuppositions* as well as by *refocusing* the course of action, which they called “moving away.” Moos and Spranz-Fogasy (2024) focused on a specific questioning technique, that is, a formulation combined with a hypothetical question, and its potential to elicit self-reflection in clients. Neither of these studies, however, focused on a particular type of question.

This study focuses on the sequential organization of QfSG in coaching. More specifically, it investigates clients’ responding activities to wh-questions in their entire range of possible reactions, that is, their response possibility space (Stivers, 2022). This is particularly interesting for wh-questions, which allow for a variety of (extended) responses. We thereby consider the degrees of (un-)cooperativeness along a continuum of (dis-)aligning and (dis-)affiliative responses. The following research questions guide the analysis: **How do clients respond to wh-questions for solution generation in coaching? To what degree do their responses orient to or engage with the question’s agenda and/or the underlying activity? How (un-)cooperative are coaching clients in their immediate reactions?**

## Method, data and process

**Method:** We use CA (Schegloff, 2007; Sidnell, 2010; Sidnell and Stivers, 2013) to investigate coaching clients’ responding activities immediately following QfSG. According to CA, the practices under scrutiny emerge in and are organized by the communicative, interlocked behaviors, that is, the (re)actions, of the participants and their mutual and collaborative illustration of what is happening in the interaction (Janusz and Peräkylä, 2021). CA constitutes a particularly suitable method to investigate authentic questioning sequences in their local emergence, in their sequentiality, as well as in their (un-)successful contribution to change/solution generation.
**Data:** Data stem from a larger corpus of German systemic, solution-oriented business coaching interactions collected between 2021 and 2023 within the QueSCo project (Graf et al., 2023, 2024). The QueSCo corpus consists of 14 dyadic, online and face-to-face processes with 50 sessions (approx. 62 hours of data). These processes were recorded by the coaches and originally transcribed according to cGAT2 minimal transcription conventions (Schmidt et al., 2016; extracts in this article were adapted to CA transcription conventions (Hepburn and Bolden, 2013)). For this study, four coach-client dyads were randomly selected to represent the range of the collected material and to help reveal patterns of clients’ responding activities that go beyond specific clients. They include two male (CO5 and CO7) and two female coaches (CO3 and CO10) with three female clients (CO3_CL1, CO5_CL1, CO7_CL1) and one male client (CO10_CL1) of different ages and professional backgrounds.^3^ The processes include 11 sessions, 5 face-to-face and 6 online, amounting to 12.5 hours of recorded data.
**Process:** Building on the QueSCo typology (Graf et al., 2024), QfSG (i.e. questions topicalizing ideal solutions, resources, hindrances and strategies as well as questions evaluating interim results) were identified in the four selected processes. Questions topicalizing hindrances, however, were excluded due to their limited number and proximity to problem-oriented questions. Next, all questioning sequences initiated by wh-questions (both telling and specifying) were collected. Multi-unit questioning turns (Linell et al., 2003), that is, coaches’ turns that included more than one distinct and conditionally relevant question, were excluded as this influences clients’ responding behavior. This resulted in a collection of 122 wh-questioning sequences with a focus on solution generation. Table 2 gives descriptive statistics of the distribution of these wh-questions across the four processes:

**Table 2.** Overview of the distribution of wh-questioning sequences across four processes.

Process	Session 1	Session 2	Session 3	Total # of wh-sequences
CO3_CL1	19	13	5	37
CO5_CL1	4	17	8	29
CO7_CL1	13	9	16	38
CO10_CL1	12	6	/	18
Total				122

An in-depth, case-by-case inductive analysis of clients’ turns that immediately followed the question was carried out by Melanie Fleischhacker.^4^ Using CA to uncover patterns, collections of responses were built thereby also drawing on prior linguistic work (e.g. Dionne et al., 2024; Mack et al., 2016; MacMartin, 2008; Muntigl, 2024; Stivers, 2022). The emerging patterns were discussed amongst all authors in data sessions. Fleischhacker iteratively re-analyzed the 122 sequences until stable categories were identified.

## Findings

The analysis yielded five recurrent categories that describe clients’ responding activities to wh-questions with solution-oriented agendas in both form and content in coaching, paying particular attention to both the progressivity of the sequence and the ongoing solution-oriented course of action. Organized according to an increasing degree of cooperativeness (i.e., alignment and affiliation) these include: “no(n) answer”, “(other-initiated) repair initiation”, “not answering and refocusing”, “answering” and “extended answering.” Table 3 shows the frequency of the categories. Though the differences between specifying and telling wh-questions were considered for the categorization of responses as either doing “answering” or “extended answering” (see below), they did not have an influence on the responding activities or (un-)cooperativeness of clients in general.^5^

**Table 3.** Frequency of response categories within the processes and across the entire data set.

Category	CO3_CL1	CO5_CL1	CO7_CL1	CO10_CL1	Total # of wh-sequences
No(n) answer	4	4	3	5	16
Repair initiation	7	0	3	2	12
Not answering and refocusing	2	3	1	4	10
Answering	8	12	7	5	32
Extended answering	16	10	24	2	52
Total					122

While the analysis revealed a continuum of (non-)cooperative reactions, coaching clients display an overall willingness to cooperate, that is, to engage in the solution-oriented agenda of the question: “answering” and “extended answering” make up 84 out of 122 or 68.9%, of responses. Also, a fine-grained analysis of the reaction categories “repair initiation” and “not answering and refocusing” evinced further elements of cooperation or affiliation. Discussing a selection of examples from the data, the goal of the findings section is not only to illustrate the categories but also to showcase how coach and client actually “do solution work” together. To address existing research gaps, the focus here will be on more cooperative client actions. For this reason, the “no(n) answer” category, where clients either remain silent or provide a non-answer, that is, a no access response, thereby entirely blocking the progressivity of the sequence will not be addressed (but see Dionne et al., 2024).

### (Other-initiated) repair

This category includes responding activities that lead to immediate repair by the coach: mere acknowledgment of the question (e.g. “hm”) and (predominantly) other-initiated repair involving problems of understanding. Such non-answers (e.g. Stivers, 2022) do not provide the “sequentially-fitted next” (Kitzinger, 2013: 242) thus delaying the response. This disrupts the progressivity of the sequence and suspends the ongoing action. Thus, they are often considered strong forms of disalignment (Stivers and Hayashi, 2010) or as resisting actions (Muntigl, 2024). Other-initiated repairs can be related to problems of hearing, speaking or understanding (as in the instances here) or they may signal upcoming dispreferred responses (e.g. challenge or disagreement; Pomerantz, 1984) or other forms of disaffiliative action (Kitzinger, 2013; Schegloff, 2007). However, the latter is less frequently the case here. Rather, clients try to cooperate by indicating trouble-sources (e.g. problems with scope or reference) often readily providing candidate understandings and thus being (at least in part) affiliative and cooperative. Such indications help the coaches adjust their previous turns accordingly. In this vein, repair work ensures that “the interaction does not freeze in its place when trouble arises, that intersubjectivity is maintained or restored, and that the turn and sequence and activity can progress to possible completion” (Schegloff, 2007: xiv). By initiating repair or producing acknowledgment tokens, clients minimally orient to the wh-question and its agenda. This is the case in 12 out of 122 sequences. Excerpt 1 provides an example of an affiliative repair initiation.

**Excerpt 1: CO3_Cl1_S2:**

1
**CO3**
und sie solltn sich vorstelln=oder wir ›zwei ›wir tun jetz mal so‹
*and now you should imagine=or the two of us ›we pretend for now‹*
2wir sitzn im kino. (.) ja [und (.)] es (.) jetz
*we are in the cinema. (.) yes [and (.)] the (.) now*
3
**CL1**
[ hmhm ]4
**CO3**
£geht der vorhang auf,*the curtain opens*,5(0.4)6
**CO3**
.hh und jetz kommt der (.) äh film äh (.) lenas leben,.*hh and now comes the (.) uh movie uh (.) lenas life*,7
**CO3**
[im j]ahr zweitausndsechsundzwanzig£.
*[in the y]ear twothousandtwentysix£.*
8
**CL1**
[hmhm]9(0.7)10
**CO3**
.h (0.7) was mein sie °was sehn wir da°,.h (0.7) *what do you think °we see there°*,11(3.4)12 →
**CL1**
also was ich gern sehn wü:rde [hhuh Huhh] ä::m (.) wä::r (2.7)
*well what i would li:ke to see [huhh Huhh] u::hm(.)would be:: (2.7)*
13
**CO3**
[£ ja][£ yes]14
**CL1**
auf alle bereiche jetz bezogn oder auf die beruflichn vor allm.*related to all areas now or to the professional ones especiall*y.15(0.9)16
**CO3**
äh ich würd alle einbeziehn,‹[ja, weil das] is teil ihres lebens,=
*uh i would include all,‹[yes, because that] is part of your life,=*
17
**CL1**
[ hmhm ]18
**CO3**
un[d diese]s ganze umfeld.=sie könn [das ja] nich abspaltn.=
*an[d thi]s entire environment.=you cannot separate [that].=*
19
**CL1**
[ ja ][ yes ]20
**CO3**
=denk ich (.) aber der fo:kus (.)und da °f führ führe-° (0.2)
*=i think (.) but the fo:cus (.)(nd there g gui guid- (0.2)*
21
**CO3**
[.hh da gehnwa schon hin. (.) [ja (.)] ähm (0.7) sollte
*[.hh that’s where we do go. (.) [yes(.)] uhm (0.7) should be*
22
**CL1**
[ja (.) genau (.) ja
*[yes (.) exactly (.) yes*
23
**CO3**
auf dem berufen- ruflichn natürlich sein, [. . .]*should be on the profession professional naturally*, [. . .]

In this extract, coach and client engage in projecting ideal future (professional) scenarios (see “questions topicalizing ideal solutions”). After goal-agreement, they moved on to this first, solution-oriented activity. In the question preface, the client is prompted to think of a hypothetical scenario (see e.g. Weatherall and Gibson, 2015): sitting in a movie theater watching her life in 5 years. This scenario is encouraged with the direct request “you should imagine” and the collaborative immediate self-repair “we pretend” (line 1). In the stepwise introduction, the coach also conveys a positive mood indicated by her smiley voice (in lines 4–7). Dependent on this hypothetical horizon (Peräkylä, 1995), the wh-question makes relevant a description of what the client imagines her life to be. The client produces positive feedback particles thus accepting this ideal, hypothetical scenario. A long gap of more than 3 seconds ensues in which the coach withholds from taking a turn.

In line 12, the client starts her answer with a well-preface, indicating a complex, non-straightforward response (Schegloff and Lerner, 2009). Another indicator of potential trouble is the type-nonconforming response to the telling question (Thompson et al., 2015), which repeats part of the question and does not immediately describe the envisioned future. Rather, the client changes “what we see there” (line 10) to “what I would like to see” (line 12) as common frame of (hypothetical) reference while still orienting to the ideal scenario projected by the coach’s question. Her emphasis on “would like” and the change of the collective “we” to the singular pronoun “I” makes clear that this is her (subjective) scenario thereby claiming epistemic authority (Stivers, 2022). This is followed by mitigating, affiliative laughter, which the coach echoes by way of a smiley voice and an accepting “yes” that elicits further talk and confirms the slightly modified framing (line 13). However, the progressivity is still halted as the client produces a delaying “u::hm” and a stretched “would be::” followed by a pause, alerting to the possibility of repair yet holding the turn (Kitzinger, 2013) and suggesting thinking.

The client then breaks off mid-sentence to initiate repair and clarify her understanding of the question. She indicates the trouble source by asking an alternative question (Drake, 2021) that addresses the scope of the wh-question: should it include all areas of life or focus on the professional realm (line 14)? The two alternatives represent candidate understandings of the scope (Linell et al., 2003). By asking for this specification, the client indicates her willingness and commitment to eventually answer the question (correctly), even though she cannot do so immediately (Bolden, 2010). Additionally, the turn-final placement of the professional focus indicates preference over the first and demonstrates knowledge of coaching interactions, that is, of “doing coaching” which is geared toward professional rather than personal matters. This way, the client still shows cooperativeness with the agenda and the institutional expectations.

In response, the coach – as is conditional for alternative questions – selects one candidate understanding “include all areas” (Linell at al., 2003; line 16) and provides an account for said choice (lines 16–20). Nevertheless, in her repair solution, she does confirm that the eventual focus will be on the client’s professional life (lines 20–23). After the scope of the question has been clarified, the client starts imagining her future (not in the excerpt). Solution generation is suspended yet quickly resumed.

### Not answering and refocusing

With “not answering and refocusing”, clients respond, but do not produce a sequentially fitted next pair that engages with the projected action. Such “answer-like responses” (MacMartin, 2008) may superficially affiliate and align. But rather, with this “moving around” (Dionne et al., 2024) or “refocusing” (MacMartin, 2008) action, clients shift the course of action proposed by the wh-question toward their own, alternative course of action (see also evasive answers (Heritage and Clayman, 2010) or transformative answers (Stivers and Hayashi, 2010)). In this way, “clients may effectively sidestep, bypass, or circle around courses of action, question constraints, or problematic elements thereof” (Dionne et al., 2024: 18) without interrupting the progressivity of the sequence. This might entail focusing on or looping back to (further) problem talk, which suspends solution generation for the time being. In this case, “not answering and refocusing” can be seen as a resistive response (Dionne et al., 2024) in which clients disalign and disaffiliate with the question and the overall course of action and are little cooperative. Yet, clients can also introduce new topics or produce (further/alternative) solution talk thereby aligning/affiliating with the underlying solution-oriented agenda even though they do not align with the question. Following Clayman and Heritage (2002: 247), solution-oriented refocusing responses fall into the positive dimension of resistance by moving “beyond the parameters of the question, producing actions or addressing topics that were not specifically called for.” A refocusing yet solution-oriented and in this sense cooperative example is given in excerpt 2. There are 10 cases of “not answering and refocusing” in the data.

**Excerpt 2: CO3_Cl1_S2:**

1
**CO3**
wo könnten sie das?
*where could you do that?*
2(1.7)3 →
**CL1**
°in_nem neuen (.) job.° hh HH huh hum .hhh ähm (.) und ja: (.)
*°in a new (.) job.° hh HH huh hum .hhh uhm (.) and ye:s (.)*
4ich (.) wie gesagt=ich (.) glaub irgend_ne art von
*i (.) as i said=i (.) think some kind of*
5weiterbildung irgend_ne art von: ›ja genau‹ ›irgend_ne art
*further training some kind of: ›yes exactly‹ ›some kind of*
6weiterbildung glaub ich‹ .hh schafft nochmal bisschen (0.7)
*further training i think‹ .hh provides again a bit (0.7)*
7mehr (.) fundier:ung. (.) in n in dem sinn (.) ›also klar‹
*more (.) foundati:on. (.) in n in the sense (.) ›well sure‹*
8(.) hilft_s mir auch,=das is (.) gar keine frage.=aber ich
*(.) it helps me too,=that is (.) out of the question.=but i*
9hab scho:n das gefühl dass ma .hhh (0.7) dass (.) äh (.)
*have actua:lly the feeling that one .hhh (0.7) that (.) uh (.)*
10das (1.2) das syschtem in deutschland (.) ja schon so isch
*the (1.2) the system in germany (.) is actually like this*
11dass wenn ma (.) was vorweisen und auf den tisch legen kann
*that if you (.) can show and put something on the table*
6 lines omitted12.hhh [was] ich jetzt persönlich (0.6) .h ›irgendwie.*hhh [which] i now personally (0.6) .h ›somehow*13
**CO3**
[hmhm]14
**CL1**
gar nich‹ s[o sinnvoll find]e, (.) aber (0.5) ich glaub
*somehow do not‹ find t[hat relevant], (.) but (0.5)i believe*
15
**CO3**
[  hmhm    ]16
**CL1**
deswegen isch es schon mit (.) eine eintrittskarte.
*that is why it contributes to (.) being an entry ticket.*
4 lines omitted17
**CL1**
[wahrsch]einlich deswegen .hhh ähm (0.2) glaub ich[possib]ly that’s why .hhh uhm (0.2) i believe18
**CO3**
[ ja ][ yes ]19
**CL1**
dass das sinnvoll isch.
*that it is useful.*
(1.0)20
**CO3**
ja: (.) ›also es kann ja‹ (.) hm (.) sich da hm ne (.)
*ye:s (.) ›well it can well‹ (.) hm (.) be hm yes (.)*
21ne eigene °erfahrung einflechten°,*interweaved °with own expertise°*,22
**CL1**
ja (.) gena[ u ].
*yes (.) exactl[y].*
23
**CO3**
   *[ahem]*24
**CO3**
ähm (0.3)((schnalzt)) (.) es is oft so (.)
*uhm (0.3) ((clicks tongue)) (.) it is often like that (.)*
25zum beispiel auch bei öffentlichen ausschreibungen
*for example also with public job advertisements*
26[dass das] explizit nachgefragt [wird.]=welche ausbildung
*[that it] is explicitly asked [for.]=which trainings*
27
**CL1**
[ hmhm ]        [hmhm]27
**CO3**
haben sie als coach gemacht, . . .
*have you done as coach, . . .*

Prior to the wh-question on specific strategies, the coach asked the client to estimate her progress toward the agreed-upon goal and to think about concrete next steps to reach it. She first responds vaguely, mentions “gathering more experiences” as a next step and (reacting to the specifying wh-question “which kinds”) adds working with different target groups as one way of acquiring new skills. Without question preface and in direct reference to this, the coach asks another specifying wh-question in line 1, making concrete locations or possibilities (i.e., solution strategies) for expanding the target group conditionally relevant (see “questions topicalizing solution strategies”).

After a gap of almost 2 seconds, which potentially foreshadows disalignment, the client first produces a type-conforming response “in a new job” (in line 3). However, the quiet delivery and the ensuing laughter particles suggest this to be a nonserious, sarcastic or joking response (MacMartin, 2008; Potter and Hepburn, 2010) that does not actually engage with the solution-generating agenda of the question. It only constitutes a pro-forma answer.

Since the coach does not intervene, after the hesitation marker “uhm”, the client refocuses on the previous question instead and lists further education as another possible next step (starting in line 4). The refocusing is indicated by the conjunction “and” – suggesting an extension of prior talk (Stivers, 2022) – and the meta-pragmatic comment “as I said”. The client then reflects on partaking in further education programs as means to getting “more foundation” (line 7) and as possible solution strategy. She justifies this as a possibility to not only gain more experience but also to play by the rules of the system in which being able to “put something on the table” (line 11) particularly matters thereby further stressing the relevance of her solution alternative. The client is thus seeking concrete solutions to enhance her chances on the current job market. Even though she steers away from the coach’s interactional project, that is, disaligns with the question, marking the coach’s current course of action as to some degree irrelevant for her concern or current state of mind (Dionne et al., 2024), she still engages in solution generation and responding. In this way, she affiliates with the solution-generating project and shows cooperativeness in this regard. With her alternative course of action, she asserts primary rights over her concerns while being open to exploring her experiences and possibilities as suggested by the coaches’ questions.

In line 20, the coach affiliates and affirms the client’s final assessment. She further supports her client’s claim and uses the clients’ alternative course of action as a new way forward (not in the excerpt).

### Answering

In this category, clients perform “an action that addresses the agenda of topics and tasks posed by a previous question” (Clayman and Heritage, 2002: 242) by providing the projected second pair part. The conditional relevance of the question is fulfilled; the progressivity and contiguity of the ongoing talk are maintained (Schegloff, 2007). The topic, agenda, form and action made relevant by the question are considered in syntactically matching responses, that is, clients align and affiliate. For specifying wh-questions this entails providing the piece(s) of information sought for; for telling questions, this means providing extended, multi-unit responses without going beyond the scope of the question. When clients do “answering” they thus respond to what the question is looking for and orient to the solution-oriented agenda, yet they do not engage in further (self-)reflection, explanation or elaboration. The action of the question is fulfilled, no more and no less even though responses vary considerably in length (see also e.g. unelaborated answering, Stivers and Heritage, 2001) especially in the case of telling questions. Due to the focus on solution generation, the differences in responding to specifying questions (Fox and Thompson, 2010; Thompson et al., 2015) are not considered here.^6^ The notion of preferred versus dispreferred answers is not useful either since the rejection of (proposed) solutions, solution alternatives, etc. also contributes to solution generation and orients to the agenda of the question. With 32 out of 122 sequences, the “answering” category is the second most frequent in the data. Excerpt 3 first gives an example of “answering” to a specifying wh-question, while excerpt 4 features an answer to a telling wh-question.

**Excerpt 3: CO7_CL1_S3: Specifying wh-question**

1
**CL1**
wie viel von dem plan in summe es war, (1.5) vielleicht vierzig
*how much of this plan it was in sum, (1.5) maybe forty*
2prozent. (0.5) >£[definitiv] zu (.)zu wenig um in meiner
*per cent. (0.5) >£[definitely] too (.) too little to be in my*
3
**CO7**
[ mmh ]4
**CL1**
komfortzone zu sein.£< (.) [.hh (.)]
*comfort zone.£< (.)[.hh (.)]*
5
**CO7**
[ja ja]
*[yes yes]*
6
**CO7**
ja [ja] .hh (.) a:ch sie sagn so vierzig prozent zu wenig, .h (0.2)
*yes [yes] .hh (.) o:h you say forty per cent too little, .h (0.2)*
7
**CL1**
[u:hm]8
**CO7**
wo würde die komfo:rtzone anfangn für sie?
*where would the comfo:rt zone start for you?*
9(3.3)10 →
**CL1**
sechzig, siebzig,*sixty, seventy*,11(0.5)12
**CO7**
hmhm (.) hmhm (.) hm (0.9) ja: (.) verstehe (0.3)[. . .]*hmhm (.) hmhm (.) hm (0.9) ye:s (.) i understand (0.3)*[. . .]

Prior to excerpt 3, coach and client had initiated solution generation after the client’s troubles telling concerning a difficult period in her professional life as an executive, aggravated by the lack of a plan or strategy. Orienting toward a prospective solution, the coach then asked how much of a plan might already be in place. In line 1, the client answers this question by providing a quantitative estimate “maybe forty percent”. She immediately qualifies this as “too little to be in my comfort zone” (lines 2–3) expressing her emotional stance. This is accompanied by a smiley voice bordering on laughter which may downplay the complaining nature (Potter and Hepburn, 2010) yet possibly also her own vulnerability and helplessness. In a transforming action (see Graf et al., 2024: 62), the coach again steers the interaction toward solution generation by focusing on the client’s comfort zone in his specifying wh-question (line 8). This question can be seen as either initiating the topicalization of client resources by focusing on prior positive situations and experiences to be built on for solution generation or as a question that topicalizes an ideal state of mind.

A gap of 3.3 seconds ensues but is not treated as problematic since pre-beginning non-verbal behavior (the client is squinting her eyes and tilting her head from one side to the other) suggests a calculating or thinking action (Stivers, 2022). The client then produces a phrasal response “sixty seventy” in line 10 quantifying how much of a plan would be necessary for her to feel comfortable.

The coach first receives this with minimal acknowledgment tokens and then with an upgraded “yes” and an expression of his epistemic stance “I understand” (line 12). By orienting to the action and content of the question, addressing an ideal state of mind, the client shows her cooperativeness to engage in the solution-oriented project as this possibly indicates where the coaching interaction should lead.

**Excerpt 4: CO10_Cl1_S1: Telling wh-question**

1
**CO10**
.hh wir bei:de (0.5) treffn uns am fünfzehnten april,.*hh the two: of us (0.5) meet on fifteen of april*,2(0.3)3
**CL1**
hmhm4
**CO10**
nächstn jahr[es].
*of next yea[r].*
5
**CL1**
[hmhm]6(0.3)7
**CO10**
((schmatzt)) (1.0) und sie kommen
*((smacks lips)) (1.0) and you come*
8mir freudestrahlend entge[gen],*radiant with joy towards [me]*,9
**CL1**
[hmhm]10(1.3)11
**CO10**
[und sagen me::ine gü:te
*[and say my:: go:d*
12
**CL1**
[›und ich sage‹ frau berg
*[›and i say‹ mrs. berg*
13
**CL1**
sie werdn ((klatscht)) es nicht glau:ben=
*you will ((claps hands)) not belie:ve it=*
14
**CO10**
=es ist wirklich (.) un(.)glaub(.)lich mir geht_s (.) so gut
*=it is really (.) un(.)believe(.)able i am feeling (.) so good*
15es könnte mir besser gar nicht [ge]hen,*i could not be feeling any [be]tter*,16
**CL1**
[ja][yes]17(0.2)18
**CO10**
was erzähln sie mir.=
*what do you tell me.=*
19 **→**
**CL1**
=.h sie werdn_s nicht [glau:ben .h wir haben jetzt tatsächlich
*=.h you will not [belie:ve it .h we now actually*
20
**CO10**
      *[((CO is moving papers around))*21
**CL1**
noch ne vollzeitkraft für die zahlungen bekomm,*have gotten another full-time employee for billing*,22
**CO10**
hmhm23
**CL1**
↑und (1.4) hatten ein projekt gestartet das ging reibungslos
*↑and (1.4) we have launched a project that went so*
24über die bühne=dess wir jetzt eine onlinebasierte (0.3) ba
*smoothly=so that we now have an online-based (0.3) ba*
25datenbank haben,*database*,26(0.2)27
**CL1**
wir haben diese alte access datenbank äh
*we have thrown this old access database uh*
28((druckst)) (.) i in die in die müllgrube getragen,*((stammers)) (.) i into the trash pit*,
*3 lines omitted*
29
**CL1**
=und sie werdn_s nich glauben ich wa:r ((klatscht))
*=and you will not believe it i wa:s ((claps hands))*
30(0.7) im februa:r (0.2) sechs wochn in ghana. und hab da
*(0.7) in februa:ry (0.2) for six weeks in ghana. and have*
31die organisation kenngelernt=und seitdem
*become familiar with the organization there=and since then*
32läuft_s alles noch viel besser.
*everything is going much better.*
33
**CO10**
hmhm34(0.9)35
**CL1**
un hab von ghana aus gearbeitet, und .h des wa:r (.)
*an i have worked from ghana, and .h that wa:s (.)*
36es war einfach gold wert.
*it was worth a mint.*
37(3.3)38
**CO10**
hmhm39(0.9)40
**CO10**
was toppt des ganze noch?
*what tops all of that?*

In excerpt 4, coach and client are working on ideal solutions to arrive at potential goals for the coaching process. In such contexts, (hypothetical) questions for solution generation (i.e., “questions topicalizing ideal solutions”) are typical and frequent. Here, the client is asked to imagine a hypothetical, future situation in which he meets the coach and tells her about positive developments. Before the actual wh-question in line 18, a telling question that elicits a narrative of radically positive changes in the client’s life and emotional wellbeing, the coach carefully introduces the hypothetical scenario. Interspersed with pauses (e.g. in line 2 and 7), which leave space for the client’s acknowledgment, the coach engages in a stepwise introduction of the hypothetical activity (Peräkylä, 1995; Weatherall and Gibson, 2015). To prompt the scenario, she first refers to a future date (lines 1–4), then describes the client’s emotional state (lines 7–8) and uses animated talk to portray the client’s joyful exclamation and mood (lines 11–15). Using the client’s voice in a quoted dialogue suggests closeness (Moos and Spranz-Fogasy, 2024: 4) and direct access to feelings and thoughts (Weatherall and Gibson, 2015). This step-by-step introduction provides necessary background information for the client’s responding activity.

The client produces various affirmative feedback particles signaling acceptance of the hypothetical scenario. In fact, the client already starts imagining the hypothetical future encounter in overlap with the coach’s question preface in line 11. Here, the client provides an extension of the coach’s mood description switching almost naturally into a first-person dialogue “and I say”. The structural parallel in the overlap in lines 11 and 12 (“and say”/“and I say”), the latching between lines 13 and 14 (when the client concedes speaking rights to the coach) as well as the mirroring of words (“you will not believe it” – “it is really unbelievable”) suggests a true “moment of meeting” (Herrera et al., 2023). Such co-constructed utterances index a high degree of cooperation, solidarity and involvement. The client has already understood the projected task, which is made conditionally relevant by the wh-question. The cooperative client behavior is also illustrated by the immediate reply starting in line 19. In accordance with the telling-question, he gives a multi-clause response listing four genuinely positive professional developments (another employee, a new data base, a visit to Africa and doing remote work) using the conjunction “and” (lines 23, 29, 35), while the coach engages in active listening (Fitzgerald and Leudar, 2010). In his answer, the client also fully engages in the imaginary scenario and dialogue with his coach by repeating the introductory phrase “you will not believe it” in which he also directly addresses the coach (lines 19 and 29). The clapping in line 29 and the positive assessments (line 32 and 36) suggest that the client has taken on the enthusiasm and good mood as well. Overall, the client successfully engages in solution generation and is fully cooperative. Though he delivers his story in various units, it does not go beyond the scope of the question and stays within the confines of the hypothetical scenario.

The coaches’ continuer in line 38, which follows the 3 second gap in line 37 after the client’s final assessment, can be read as another attempt at eliciting further talk (see expandable response, Muntigl and Zabala, 2008). Since the client does not self-expand, the coach poses another wh-question.

### Extended answering

As in the previous category, clients align and affiliate by providing the corresponding second pair part (to telling or specifying wh-questions). However, here clients also respond to the inherent potential of questions to induce (self-)reflection and elaboration (Köller, 2004) as well as to the implicit call to move beyond the agenda of the question (see Läpple et al., 2021). This is vital for change and transformation (Muntigl and Zabala, 2008). Extended answers go beyond the scope of the question and requested action – irrespective of specifying and telling questions – as clients reflect or elaborate on the underlying idea(s) or presupposition(s), respond to topic proffering, narrate stories, provide examples and illustrations or comment on, account for, problematize or assess their own responses, thereby often exploring various (new) topics, emotional or epistemic states. Their extended turns-at-talk thus include the answer to the initial question as well as an expansion component in which clients voluntarily provide additional information on their life, thinking, feeling, behavior, etc. (Stivers, 2022: 150). Similarly to Stivers and Heritage’s (2001) findings in doctor-patient interactions, such expansions can entail rather brief elaborations or complex and extensive narratives that allow clients to move further away from the question and potentially introduce their own (interactional) projects. Again, no distinction is made between preferred and dispreferred responses here. However, “extended answers” display different degrees of (dis-)continuity with the solution-oriented wh-question (see also Heritage and Clayman, 2010) with clients either expanding on solution-focused talk, oscillating between solution- and problem-oriented talk or shifting into troubles telling. The expansions are only minimally prompted beyond the question itself, for example, via active listening and continuers. In any case, clients orient to the solution orientation proposed by the question (at least during part(s) of their expansion) and the change project may thus be moved forward in the coaches’ third position. With 52 out of 122 sequences, this category is the most frequent. The following extract illustrates a client’s extended answering that engages in further solution talk beyond the scope of the question.

**Excerpt 5: CO5_CL1_S1: Extended solution orientation**

1
**CO5**
.hh und wie könntest du es denn leichter machen die seite.*hh and how could you make it easier for you to*2dann (.) auch in dem kontext zu zeigen, dass es nicht
*then (.) show this side also in this context, so that it*
3so viel kra:ft kostet.
*doesn’t take so much e:ffort.*
4(1.1)5 **→**
**CL1**
ja wahrscheinlich das bewusstwerden=und das ist jetzt
*yes probably the awareness=and this is now*
6gerade was ich (.) was jetzt gerade passiert.
*at the moment what i (.) what is now happening.*
7(0.7)8
**CO5**
hm9
**CL1**
aso m m mir bewusst zu sein (0.4) wenn ich in diesen raum
*so to b be aware (0.4) when i am entering this room*
10trete muss diese seite aktiv werden,*this side has to become active*,11(0.4)12
**CO5**
°ja°
*°yes°*
13
**CL1**
ist sicher besser als im raum zu sein, und jedesmal
*is definitely better than being in the room, and every time*
14überrascht zu sein, dass es jetzt (.) diese seite braucht,*being surprised, that it is now (.) that the side is needed*,15und dann >oh shit warte jetz [mal]< und dann beginnt des .h
*and then >oh shit wait now a [sec]< and then this starts .h*
16
**CO5**
[hm]17
**CL1**
und [das] kostet mehr energie als vorbereitet (0.7) in den
*and [that] costs more energy than entering (0.7) in the*
18
**CO5**
[ja ][yes]19
**CL1**
raum zu treten und zu wissen (0.8) j jetzt ist diese
*entering the room and knowing (0.8) n now this side*
20gefragt. (0.7) und jetzt geh ich mit dieser seite schon
*is called for. (0.7) and now i am entering with this side*
21
**CL1**
direkt von anfang an rein.
*directly from the very beginning.*
5 lines omitted22
**CO5**
und dass die seite jetzt auch gebra:ucht wird, und .hhh
*and that the side is now also nee:ded, and .hhh*
23(.) dass die seite auch zu dir gehört.
*(.) that the side is also a part of you. (. . .)*

In the session under scrutiny, coach and client have established that in difficult professional situations two opposing personality sides are at play within the client. A vivacious “freedom side,” which seeks to express itself openly and creatively, and a structured “managing side,” which is less spontaneous and expresses itself calmly and clearly. In a formulation prior to this extract, the coach summarizes the clients’ conflict: using the managing side is crucial to be heard amongst older colleagues as a young executive but showing it costs strength and energy. This is followed by the solution-oriented wh-question in lines 1–3 that seeks for ways to more easily and strategically employ this side. In line with “questions topicalizing solution strategies”, it addresses the “how” and is geared toward a particularly challenging situation. The direct address in line 1 suggests that the client’s very own solutions are elicited.

After a gap, the client – though hesitantly – suggests becoming aware of the situation as a possible solution strategy thereby orienting to the coach’s question and its agenda (line 5). She exemplifies this by verbalizing parts of a self-alerting internal monologue (lines 5–6) and goes on to explain what she means by awareness, that is, “when I’m entering this room this side has to be active” (line 9–10). At this point, her answer can be heard as syntactically, prosodically and pragmatically complete (Moos and Spranz-Fogasy, 2024) and is positively received by the coach with a very soft “yes” in line 12. In line 13, however, the client self-expands (Muntigl and Zabala, 2008) by continuing within the established solution suggestion. She assesses her awareness strategy as “definitely better” (line 13) than her prior behavior thereby weighing solution alternatives (Graf et al., 2025a). Her prior reactions to such situations did not entail a conscious and strategic preparation, which resulted in the client being surprised by the situation, which “costs more energy” (line 17). Her surprise is again illustrated with inner monologue (line 15). The client continues to independently solidify her solution strategy by repeating her new approach, that is, “entering with this side directly from the beginning” (lines 20–21) thereby showing increasing commitment to this behavioral change and to advancing this solution project. The coach’s question thus not only generated solution strategies but has also led the client to arrive at a new, transformed stance (Moos and Spranz-Fogasy, 2024).

## The response possibility space and cooperativeness of coaching clients

**Figure 2.**
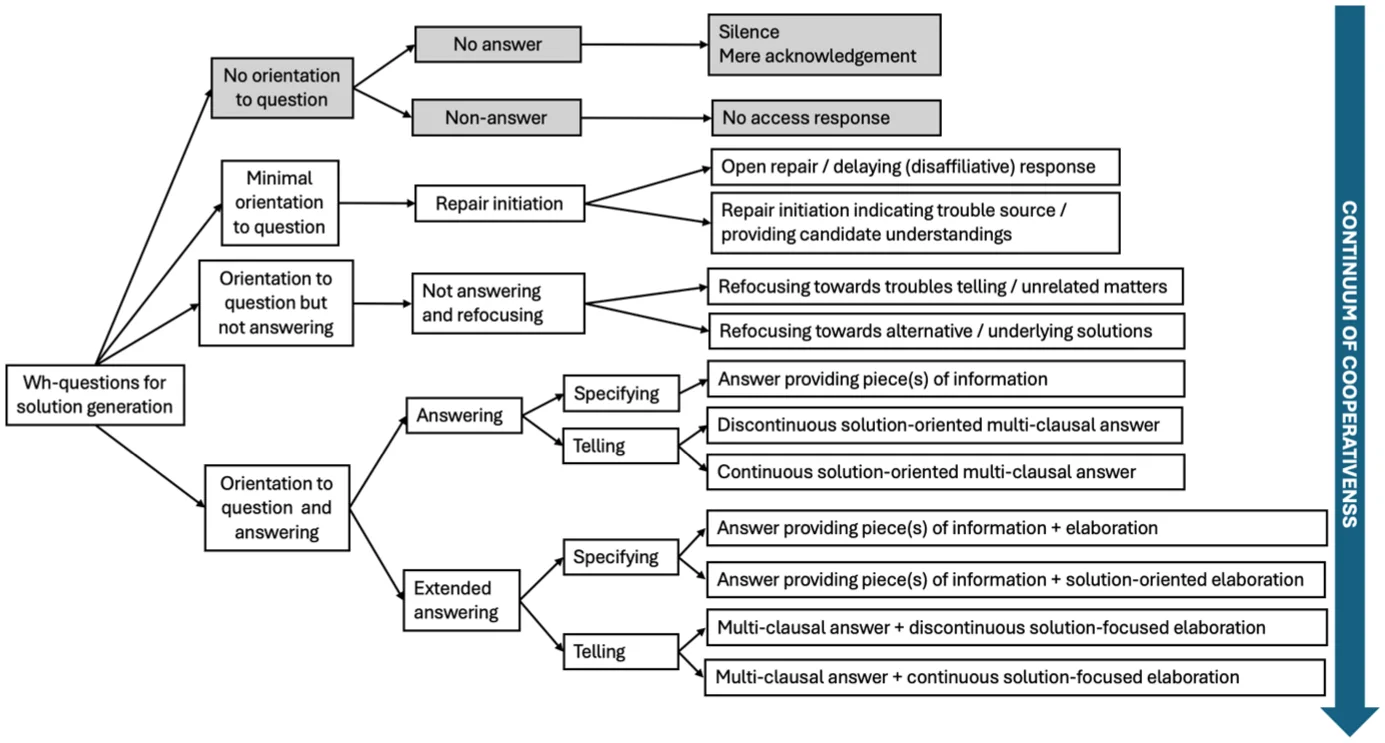
Response possibility space and the continuum of cooperativeness of coaching clients.

Following Stivers’ (2022: 175) conceptualization for polar interrogative questions, the response types – “no(n) answer”, “repair initiation”, “not answering and refocusing”, “answering” and “extended answering” – discussed above mark the **response possibility space** as well as the continuum of cooperativeness in the sense of orienting to wh-questions with a solution-oriented agenda in our data. In terms of actions and contents, responses orient to coaches’ initiating action to different degrees. Additionally, each position in the possibility space has “associated affordances and consequences that bear on the social relationship between questioner and recipient” (Stivers, 2022: 22; see also Muntigl, 2024). This affects the progress of the ongoing solution generation and the underlying change project in more or less productive ways and creates a continuum of available (dis-)affiliative and (dis-)aligning, that is, (un-)cooperative responses (see Figure 2). For instance, while responding activities can be disaligned thus interrupting the progressivity of the sequence, they may be affiliative with the solution-oriented project underlying the question and activity thus supporting the coach’s course of action.

In this sense, while disaligning via initiating repair or refocusing is, in principle, uncooperative, multiple forms of cooperation (i.e., affiliation with a solution-oriented project) are still possible (see also Stivers, 2022: 83). Repairs indicating trouble sources and including candidate understandings are more cooperative and engaging than open repairs (e.g. *What do you mean?*). Refocusing responses that address alternative solutions cooperate with the underlying solution-oriented agenda. Though the uptake of the sequentially implicated next is thus delayed or missing, clients are affiliative because they bolster the coach’s course of action (Bolden, 2010). Also, relative to the full possibility space, refocusing responses may help to avoid a no(n)-answer response or a subsequent disaffiliative action (Stivers, 2022: 87–88). Refocusing responses may also allow for continuity and progressivity of the sequence. While answers align and affiliate with the question and the solution project, that is, are cooperative, extended answers are maximally affiliative and aligning as clients also respond to the underlying request for elaboration (Greif, 2008; Muntigl and Zabala, 2008). As such, the various response types allow for degrees of (un-)cooperativeness in which clients balance (dis-)alignment, (dis-)affiliation and autonomy.

Overall, coaching clients’ responding activities within solution generation emerge as strikingly different from other helping interactions such as psychotherapy where most responses were disaligning and disaffiliative (Mack et al., 2016; MacMartin, 2008; Spranz-Fogasy et al., 2019). Frequency statistics of the response categories show that clients mostly cooperate and – as a default – provide even more information than requested. In extended answers, clients add examples, illustrations, summaries, (mini-)stories or even meta-pragmatically topicalize solutions by assessing or weighing them (see Graf et al., 2025a), for example, by juxtaposing old and new behaviors or critically reflecting on sustainable and authentic change. Additionally, our in-depth analyses revealed further affiliative behaviors, even in disaligning responses.

This remarkably high degree of **interactional cooperativeness**, that is, the engagement in solution-generating agendas and contents of questions that emerges on the interactional level, points to a particular attitude of coaching clients: they are committed, eager and willing to change; they are agentive and want to ease their (professional) problem(s) and to give up (disruptive) patterns; they voluntarily dive into self-critical and self-reflective processes and generate, discuss, assess or reject possible solutions (see also Fleischhacker et al., 2024; Moos and Spranz-Fogasy, 2024; Spranz-Fogasy et al., 2019).

Since question-answer sequences are molded by the institutional context (Heritage and Clayman, 2010), this “doing being willing and eager to change”-attitude may be rooted in the interaction type “coaching.” From such an institutional perspective, various arguments support this hypothesis. First, as mentally and emotionally functional individuals (Graf, 2019), coaching clients are invested in their own change projects during and even before the start of the coaching interaction. If coaching was suggested by managers/employers, cooperating and “doing being a good client” also means being a “dutiful” employee and being aware of the necessity to learn and develop fast. A business or academic context means that clients have learned to adopt a solution-oriented mindset and outlook on problems, for example, seeing challenges as potentials for growth. Second, clients are familiar with the interactional protocol, which is based on extensive client talk such as narrating personal and professional experiences as well as reflecting on them. As such, coaching clients voluntarily respond to the implicit pragmatic expectation for expansion in extended answers (see e.g. Mack et al., 2016; Muntigl and Zabala, 2008 for psychotherapy, and Graf, 2017 for the voluntariness of coaching clients). As envisioned in concepts like a dialogue at eye-level (Jautz, 2017) and goal-oriented self-reflection (Greif, 2008), clients thereby assume responsibility for providing the content to be worked on and for establishing the personal and emotional frame of reference that coaches can draw on (Graf, 2007). Finally, as responsible professionals, clients use extended turns-at-talk to implement their own agendas and interactional projects (see also Stivers and Heritage, 2001). By refocusing, for instance, they assume epistemic and deontic authority concerning their own change process, exerting a great(er) extent of autonomy and agency. In this way, they project an agentive self in congruence with their professional identities and construct themselves as experts of their own lifeworld (Graf, 2007). They emerge as independent interlocutors who are responsible for their own change process. By being interactionally cooperative and showing commitment to the underlying institutional agenda and responding to the expectations placed on them, coaching clients thus also emerge as **institutionally cooperative**.

**Limitations and Outlook:** Since the aim was to analyze the continuum of possible client reactions and provide a first overview, the granularity of the categories is coarse. Particularly “extended answers” need further refinement. Most importantly, the coaches’ third position should be considered next. Here, coaches decide whether to continue or abandon solution generation and how to deal with clients’ (un-)cooperative responses. Looking into the third position and beyond, that is, at longer/supra-sequential stretches of talk or even entire activities, allows to not only to investigate clients’ orientation to a single solution-oriented question but how this contributes to solution generation overall. Here, also disaffiliative or resistive responses may eventually play a critical part. Interactional and institutional cooperativeness as concept(s) should nevertheless be investigated further, as resistive behavior has dominated empirical research so far. This could be particularly rewarding in comparing problem- and solution-oriented professional helping interactions.

## Notes

1. Alignment and affiliation are considered fruitful since the usefulness of type-conformity and preference organization have been questioned in wh-questions, particularly in telling questions (Thompson et al., 2015). Also, dispreferred reaction to wh-questions for solution generation may still be affiliative as they support the underlying course of action, for example, by rejecting a solution, and allow for sequence progressivity.

2. To differentiate between the question type SOQ, which was originally developed and defined for psychotherapy, the term QfSG was introduced for coaching-specific, solution-oriented question types.

3. Written informed consent was obtained from all participants. Any potentially identifiable information has been anonymized during data transcription.

4. For the purpose of this study, neither coaches’ third positions nor supra-sequential patterns were considered.

5. From what can be gleaned from the questions only (i.e., not considering coaches’ third positions), about two thirds of the wh-questions were telling questions. This is to be expected for the professional helping format coaching that places emphasis on (self-)reflection, narratives, etc. Even so, a more in-depth look into the responding behavior of clients regarding telling or specifying questions yielded no striking difference between the two: both types of wh-questions received no immediate answer (i.e., no(n) answer, repair initiation and not answering and refocusing taken together) in about 30% of the cases and an answer (i.e. answer or extended answer) in around 60% of cases.

6. Phrase-in-clausal and clausal responses to specifying questions also orient to the solution-generating agenda and – in coaching – might also be due to the underlying expectation to expand. Single clause, phrasal or blocking responses (Thompson et al., 2015) to telling questions did not occur in this data (but see no(n) answer or refocusing responses), that is, none of the telling questions received a phrasal response only.
